# Observations upon the Importance and Value of a Knowledge of the Collateral Branches of Medicine and Surgery, in Connection with Dentistry

**Published:** 1848-04

**Authors:** James Robinson

**Affiliations:** London.


### ARTICLE II.

### *Observations upon the Importance and Value of a Knowledge of the Collateral Branches of Medicine and Surgery, in connection with Dentistry.* By JAMES ROBINSON, D. D. S., London.

(Continued from page 119.)

HAVING in our last paper shown the value and importance of pharmacy, chemistry and comparative anatomy to the dentist, and, we trust, proved how essential a knowledge of these subjects is to the scientific, if not the (pecuniarily) successful practice of his profession, we shall, in this, address our observations to a subject of paramount importance, surgery, both theoretical and practical.

The proper meaning of the term surgery is a matter of some dispute. By some it is considered to mean merely the mechanical part of physic, and that it should be confined to external and tangible affections, whether proceeding from disease or accident; but were surgery limited to this definition, cases of stone, and many others strictly surgical, would be excluded; while, on the other hand, if we take it to apply to all diseases involving a change in the organic structure of the parts, diseases of the liver and lungs, indeed, all organic diseases, in which a pathological change may be produced, would come under the care of the surgeon.

The fact, however, is, that the line of demarcation between surgery and medicine is so slight, and so difficult to be defined; the intricacies involved, so insurmountable by demonstration, that as regards the practice of the two arts, the distinction, if such there be, is, in most cases, made dependent on the convenience of the public and the inclination of the practitioner.

Nor could it well be otherwise in cases of affections of the liver; for instance, the disease may or may not involve a change of structure; if it does not, it may be, strictly speaking, said to be within the pale of medical treatment; if it does, and an operation be necessary, it at once becomes a surgical case; the same holds with respect to ascites, and twenty other complaints, in which, if the medical treatment be unsuccessful, an operation is the inevitable result.

We shall, however, take a modified view of the subject, and consider surgery to include all cases of disease or accident in which there is a change in the position or structure of the parts, not produced by constitutional disease; though, in many cases, they may produce it; but even by this arrangement the same diseases may, under different phases, become the legitimate subject of either medical or surgical treatment, or, more frequently, of a conjunction of both; thus, a case of paralysis or appoplexy produced by external violence would naturally fall under the care of the surgeon, while, if its origin were more obscure and dependent on constitutional derangement it would be confided to medical treatment.

Whatever we define surgery to be, or however we limit its application, one thing is self-evident, that a knowledge not only of anatomy, physiology and pathology, but of all the other sciences connected with the medical art generally, is essential to its scientific practice, and that whether its object be confined to a particular part as the teeth or the eye, or whether practised generally; for surgery cannot be so completely separated from medicine but that cases will arise in which an acquaintance with one or more of these sciences is not imperative, nor can the dental art more particularly be considered independent of the science and resources of surgery; for, however custom may have sanctioned the different divisions and sub-divisions of the medical art, they are all, more or less, subject to the same general principles, and the same amount—though not, perhaps, precisely the same amount—of knowledge can only insure their successful practice, and any difference that exists is more in detail than in theory. It is not necessary to the dentist, for instance, to perform the operation for lithotomy adroitly, or for the surgeon to be an expert tooth-drawer, but they should both possess a knowledge of the morbid actions that may give rise to the one operation or the other.

While, however, theoretical surgery demands a knowledge of the various sciences connected with medicine, this knowledge will be, comparatively, useless in the operative and practical portion of the art, unless the practitioner have such an amount of mechanical knowledge as will enable him to use his mechanical means in the most expert manner, and thus, as in the case of operations, lessen the time and consequent suffering to the patient.

We must not, however, be supposed to mean that lower order of mechanical tact found in the joiner or the wheel-wright; but rather those principles of animal and surgical mechanics— by means of which a dislocated arm may be reduced, or a distorted spine treated in the most scientific, and, at the same time, most successful manner; and this involves not only the selection of the best mechanical means to be employed, but the most adroit mode of using them.

Every one at all conversant with the subject, must have observed the difference exhibited by various surgeons in this branch of the art; one, for instance, performing the operation of lithotomy in two or three minutes, while another, under, perhaps, more favorable circumstances, will consume three times that amount of time. Another, reducing a dislocated arm with a tact that appears all but miraculous to those who, from want of knowledge or want of practice, have not the same facility; and which can be acquired only by an intimate acquaintance with the anatomical structure of the parts, and a thorough knowledge and perfect management of the mechanical means employed.

If, then, it is essential to the practitioner of general surgery, who, comparatively, is seldom required to exercise his skill as an operator, to acquire this mechanical knowledge and practical skill, how much more so must it be to the dentist who is hourly called upon to do so, and who, unlike the surgeon, has not time to think over and prepare for his operations perhaps days before they are to be performed, but must, in most cases, be prepared to act with off-hand promptitude.

How often is he called upon to regulate the teeth by extracting those which, by offering an opposing force, prevent the series from presenting a regular and agreeable contour; cases in which a *mistake* either in mechanical principles or physiological resource might entail permanent deformity on the patient. How often has he to treat alveolar abscess arising from the exposure of the nerve of a carious tooth. How frequently to puncture the antrum highmorianum, an operation requiring anatomical knowledge, mechanical resources, and practical skill.

All these cases require a knowledge of mechanics, and great manual dexterity, not that kind of mechanical, or rather handicraft skill of which we shall have to speak, and which is exemplified in the making artificial teeth, &c.; a branch of an infinitely lower grade than operative mechanics, inasmuch, as in the latter, anatomy, physiology, and the general principles of medicine and surgery must be brought into play before the application of the instrument to be employed.

But while a knowledge of mechanics is essential to the proper performance of operations, it would be worse than useless, unless accompanied by an adequate acquaintance with surgery and physiology. Were operations rudely performed, without reference to the powers of nature, or the resources of art, however great the manual tact of the operator, irremediable injury might be done, and cases of really slight importance converted into one of permanent deformity.

In treating of mechanics, however, it is not our purpose to divide the subject into endless ramifications of the different meanings and acceptations of the word, but to confine ourselves to those divisions more particularly applicable to dental surgery.

A knowledge of what we shall call animal mechanics, then, is as essential to the dentist as to the surgeon, and, although, perhaps, in their particular application may, generally speaking, be confined to the mouth, there is not a part of the human body, an accident to which it may be exposed, an action it may perform, or any professional service rendered it, that does not afford an illustration of some truth on natural philosophy, add to our stock of information, or from which we cannot make some deduction applicable to dental surgery.

How, for instance, could the dentist arrange with scientific certainty a set of artificial teeth, unless perfectly acquainted not only with the mouth itself, but the mechanism by which the jaws are separated or brought into contact? Thus, the lower jaw forms a lever, acted upon and moved by different muscles; the temporal and masseter muscles pulling almost directly upwards, or at right angles with the jaw, are antagonised by the digastricus and others, which assist in separating the lower from the upper jaw. We know, however, that muscles act more strongly in proportion to the straightness of their action, and less so in proportion to its obliquity, and hence, while the muscles that pull up the lower jaw exert an immense force, as may be proved by placing a substance between the more backward teeth and putting them into action; those which relax the jaw are far less powerful, and, consequently, exert a comparatively trifling amount of force. In the lion, the tiger, and the shark, the temporal and masseter muscles are still more strongly developed, and they can, consequently, crush the hardest substances without any apparent effort.

But the teeth themselves are an important consideration among the different parts of which the human body is composed, and are at the same time one of the most beautiful exemplifications of mechanical arrangement, appearing as if, severally, the fruits of distinct and miraculous agency, for it is difficult, however conversant with physiology, to imagine, how they should be formed by the laws which regulate production, or how the variety of form so beautifully adapted to the purposes for which they are intended, should be almost invariably exhibited, and thus, whether we consider the teeth themselves, or the means by which they are fixed and retained in the jaws, they are objects at once of interest, curiosity and wonder.

They may be said to constitute an extraordinary set of chisels, wedges and mill-stones, so arranged as to be most efficient for the different purposes of cutting, tearing and grinding, possessing, externally, extreme hardness, and yet having an internal organization, equal in importance and extent to that of the eye or ear, and the same powers of vitality, although their uses are purely mechanical.

It is from these circumstances, that a knowledge of the different medical sciences is essential to the treatment of these organs, and that this must be combined with an acquaintance with the mechanical laws that regulate their action when in use, and the method of supplying artificial substitutes when they are destroyed by disease or accident.

This branch of mechanics, as we before observed, differs from the handicraft of the profession, as shown in the formation of artificial teeth. The first constituting surgical mechanics, and embracing the different operations of stopping, scaling, extracting and filing, and regulating the natural teeth, or arranging artificial substitutes; while the latter, a lower branch, involves the manipulation or manufacture of the machines to be employed, such as casting models and making teeth, and adapting them to the models, making drills, &c.

From these observations it must be clear that the dental art, in its perfection, is the beau ideal of pure mechanical surgery; that its connections with mechanics are two-fold, theoretical or animal mechanics, the principles of which, when established in the mind, will be cultivated by the practice of the art, and practical mechanics, or the art of operating; and, as a celebrated writer has observed, "no art is perfect unless another art of constructing the tools necessary to the purposes of the art is embodied in it." We may add a third branch of mechanics to the two former.

The successful application of these different branches of mechanics will, of course, depend on a knowledge of these general principles, and on their especial application in practice, conjointly with mechanical ability, are an efficient knowledge of medicine, will greatly depend the success of the dental practitioner.

However much the ridicule that it has been attempted to throw on the mechanics of the profession, by some individuals possessing neither a knowledge, nor the ability to attain or apply it, we should say that a practitioner unacquainted with the laws or resources of mechanics, may be qualified to practice as a *physician* dentist, but can never, under any circumstances, fill even a mediocre position as a *surgeon* dentist.

It is clear, then, that dental surgery demands a combination of the entire branches of knowledge connected with medicine and surgery, and that, although differing from its parent stem, as to its field of action, there is no sphere in which definite and surgical mechanics can be so advantageously employed as in connection with the teeth and mouth.

